# Replacing Missing Maxillary Lateral Incisors by CAD/CAM PMMA Cantilever Bridges

**DOI:** 10.1155/2023/4302316

**Published:** 2023-07-08

**Authors:** Shayma Karray, Yosra Gassara, Emna Boudabous, Sarra Nasri, Zohra Nouira, Hayet Hajjami

**Affiliations:** ^1^Faculty of Dental Medicine, University of Monastir, Monastir, Tunisia; ^2^Research Laboratory of Occlusodontics and Ceramic Prostheses, University of Monastir, LR16ES15, Monastir 5000, Tunisia; ^3^Department of Dentistry, Fixed Prosthodontics Unit, University-Affiliated Hospital Farhat Hached, Sousse, Tunisia

## Abstract

**Introduction:**

Management of missing maxillary lateral incisors can be a challenging endeavour for dentists. Whether from agenesis or tooth loss, several treatment modalities are currently present to tackle this task to ensure satisfactory aesthetics. Most patients, especially younger patients are more likely to prefer fixed prosthodontic rehabilitation. Among these options is the computer-aided design and computer-aided manufacturing polymethyl methacrylate (CAD/CAM PMMA) cantilever bridge. *Case Descriptions.* These two clinical cases describe the management of missing lateral maxillary incisors in two Tunisian female patients with different etiologies, using CAD/CAM PMMA cantilever bridge.

**Conclusions:**

CAD/CAM technologies allow for a fairly quick and simple try-in thanks to their high accuracy as well as being predictable, minimally invasive, and affordable treatment options. This type of restoration can be put to use for mid- to long-term solutions to missing maxillary lateral incisors. However, its success depends mainly on patient selection regarding age, general health, occlusal context, and proper indication.

## 1. Introduction

In the last decade, following the rise of social media and constant online presence, patients have shown more and more demands concerning their appearance. The loss of an anterior tooth is considered an event that can hinder a patient's social life and psychological state and that needs to be addressed quickly [1, 2].

Missing upper lateral incisors can traumatically influence patients' lives, since they occupy, such as a central role, in the smile. Even young patients exhibit a need for an aesthetically pleasing smile, at all times [3, 4].

The absence of upper lateral incisors is a common condition in humans. Its occurrence can vary among different ethnic groups. This condition can be unilateral or bilateral and may occur either on its own or as part of a genetic disorder [5].

According to a 2004 meta-analysis, the mandibular second premolars and maxillary lateral incisors were revealed to be the most frequently missing across several ethnicities [6]. Tooth agenesis is usually linked to genetics as well as environmental factors, infections, chemotherapy, and radiotherapy [7].

A study conducted in Tunisia found that the prevalence of this condition among orthodontic patients was 3.6% out of a sample of 1000, according to data collected in 2018 [8].

As a dentist, managing the patient's expectations while meeting their financial needs all while quickly solving this problem can be quite a daunting task. Since its introduction in 1936, polymethyl methacrylate (PMMA) or acrylic resin has been widely used in dentistry, especially as a denture-base material [9].

The computer-aided design and computer-aided manufacturing (CAD/CAM) technologies are used for the fabrication of various ceramic restorations, including inlays, onlays, crowns, and fixed partial dentures. More recently, several researchers investigated the use of CAD/CAM technologies for the fabrication of PMMA dental prostheses and compared the materials' properties and various aspects of the conventional and CAD/CAM PMMA materials [10]. In contrast to conventional techniques, CAD/CAM quick prototyping and milling make for an attractive option in restorations.

## 2. Case Report 1

A 16-year-old healthy Tunisian female patient without any significant medical history, undergoing orthodontic treatment for maxillary lateral incisors agenesis to open the space to eventually place implant-supported crowns.

**Figure 1:**
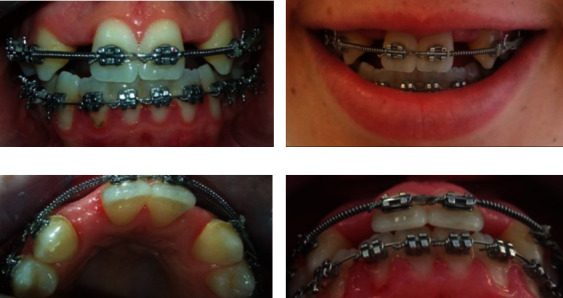
Initial clinical situation. (a) Smile line. (b) Intra-oral view. (c) Intra-oral view of anterior occlusal contacts. (d) Occlusal view of absent laterals.

During the finishing protocol, the patient was referred to the Department of Fixed Prosthodontics, for a temporary reversible and aesthetic solution to the newly opened gaps (Figure 1).

Obtained occlusion and mesiodistal spaces were favorable to placing two cantilevers PMMA resin-bonded bridges to replace both missing incisors until the patient is old enough for a definitive implant-supported restoration.

After obtaining the patient's consent, a minimally invasive preparation limited to the aprismatic enamel was done on both central incisors since there was a slight open bite. Upper and lower impressions were taken, and color choice was made using VITA Toothguide 3D-MASTER.

**Figure 2:**
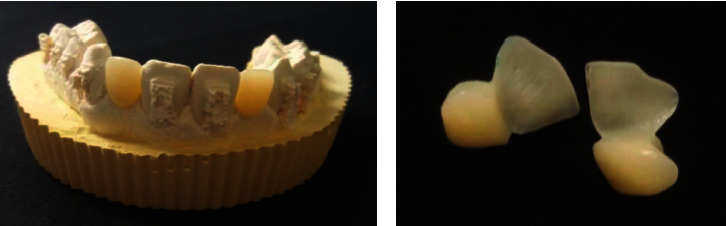
Milled PMMA cantilever bridges.

After scanning the working cast, the laboratory technician designed and milled both bridges by CAD/CAM using a PMMA block (Figure 2).

**Figure 3:**
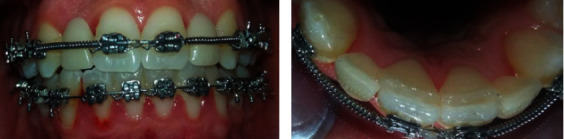
Adaptation of both cantilever bridges.

Both bridges were successfully checked (Figure 3).

Sectional isolation by the light-cured liquid dam was put in place, palatal surfaces of both central incisors were etched for 15 seconds using 37% orthophosphoric acid (N Etch, Ivoclar, Schaan, Liechtenstein), rinsed thoroughly, then a thin layer of adhesive (Tetric N Bond Universal, Ivoclar, Schaan, Liechtenstein) was light-cured for 10 seconds, and both restorations were simultaneously bonded using flowable resin (Variolink LC, Ivoclar, Schaan, Liechtenstein) of a similar shade.

**Figure 4:**
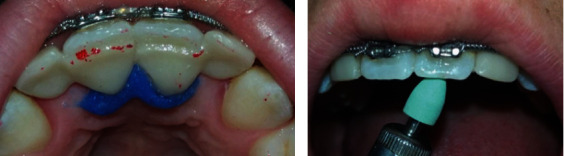
Post-bonding occlusion check.

Occlusion was checked, and final polishing was performed to ensure secure margins (Figure 4).

**Figure 5:**
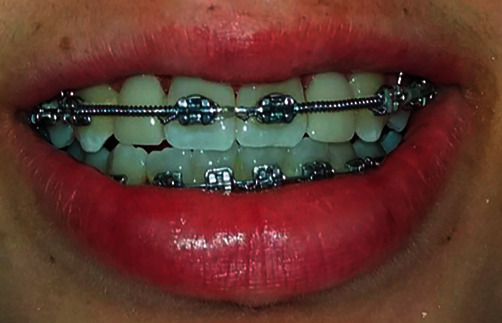
Final clinical situation.

The young patient was pleased with the final result, and the patient was instructed to maintain appropriate oral hygiene (Figure 5).

A three-year follow-up asserted complete patient satisfaction concerning her smile.

## 3. Case Report 2

**Figure 6:**
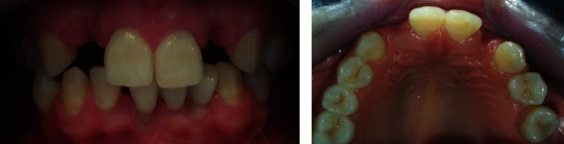
Intra-oral views of absent laterals.

**Figure 7:**
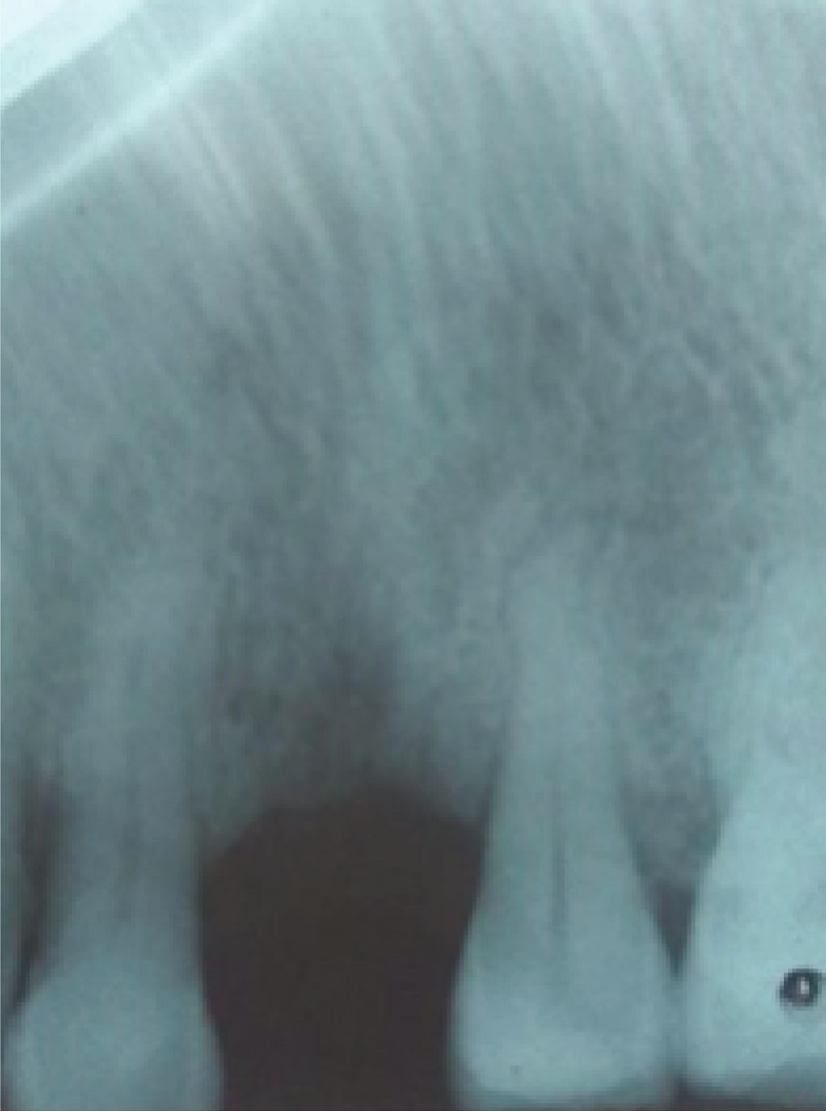
Initial X-ray showing bone loss levels in the right central incisor.

A 27-year-old Tunisian female patient with rhizomatic dwarfism, unstable type II diabetes, and chronic hypophosphatemia consulted our department to replace her missing upper lateral incisors. Upon clinical and radiographic examination, it was revealed that the patient had generalized periodontitis with widespread bone loss. The patient reported the loss of her upper lateral incisors was in fact due to periodontal disease. Both central incisors teeth #11 and #21 showed no sign of mobility with a radiological crown and root ratio of one (Figures 6 and 7). The overall occlusal context was favorable, the periodontal disease was stable, and the patient kept good oral hygiene.

Due to the precariousness of placing implants in a hypophosphatemic context, as osteointegration can be delayed, it was decided to replace each upper lateral incisor with a PMMA cantilevered bridge. The treatment plan was finalized after obtaining the patient's consent.

**Figure 8:**
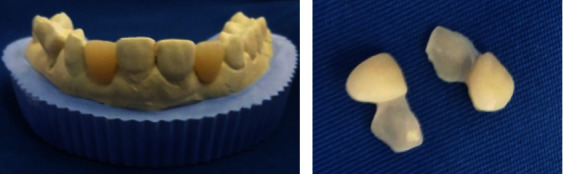
Milled cantilever CAD/CAM PMMA bridges.

Impressions with light and putty silicones were taken, and shade selection was done in a single visit. Both cantilevered bridges were designed and milled by CAD/CAM as well in the dental lab (Figure 8).

Both restorations were checked and bonded using a flowable resin (Variolink LC, Ivoclar) after proper isolation. Finally, occlusion control and final polishing were done.

**Figure 9:**
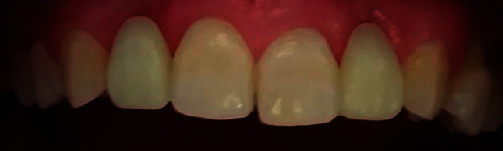
Intra-oral view post-treatment.

**Figure 10:**
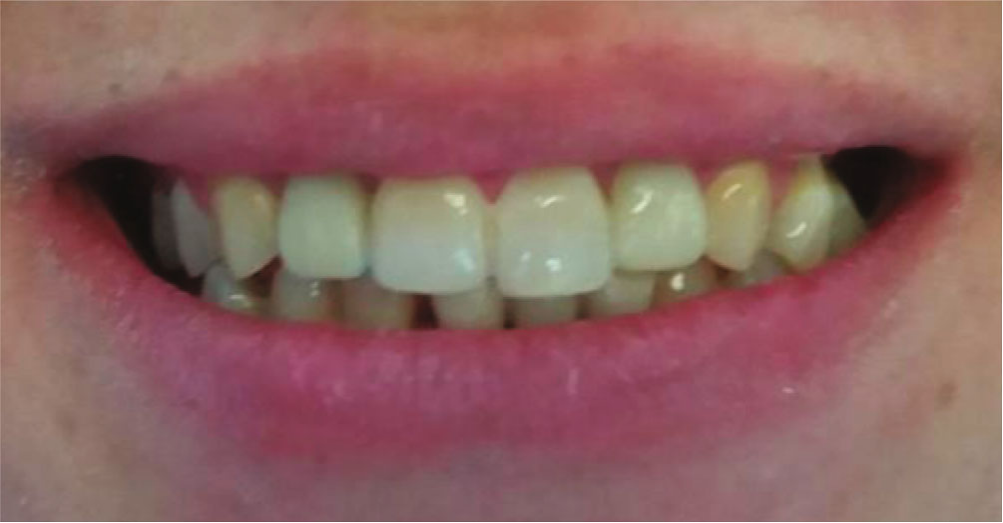
Frontal view of smile post-treatment.

The patient has been followed for 3 years, and the patient has so far maintained treatment results (Figures 9 and 10).

## 4. Discussion

For both clinical situations, replacing the missing maxillary lateral incisors with CAD/CAM PMMA cantilever bridges proved to be an effective solution. This approach provided an aesthetic outcome while adhering to the principle of tissue economy. Both patients were satisfied with the results and did not report any aesthetic or functional complaints over three years.

Rehabilitation strategies for lateral incisor agenesis can involve either opening the space and replacing the missing teeth or closing the gap and transforming the canines into lateral incisors [11]. The choice of treatment depends on the general clinical context, patient age, financial situation, and personal preference towards fixed restorations. In our first case, orthodontic treatment was used to open the gap, allowing us to place a cantilevered PMMA bridge. This approach restored the teenage patient's smile while preserving the space for a future implant-based solution.

In some cases, systemic conditions can affect the osteointegration of implants and reduce the success of prosthodontic treatment [12]. Inappropriately controlled diabetes associated with hypophosphatemia as in our second case makes the prognosis of implant-supported crowns, replacing the upper lateral incisors, quite possibly unsuccessful. Literature is scarce on the use of implant-supported rehabilitations in patients with hypophosphatemia. Due to the patient's overall health condition, we opted for a minimally invasive, low-cost solution to improve their smile.

CAD/CAM PMMA cantilevered bridges have been used as a long-term solution, even for adult patients who cannot afford implant-based or ceramic-based treatments. Both of our patients were satisfied with the final results and appreciated the quick and painless solution to their aesthetic concerns. The PMMA cantilevered bridge is a compromise from available treatment options for restoring a missing upper lateral incisor. It is preferred by younger patients over the uncomfortable removable prosthesis often associated with the elderly. Additionally, it is more conservative towards dental tissue compared with conventional full-coverage bridges and more affordable compared with an implant-supported crown. This treatment option is particularly popular due to its cost-effectiveness, simple manipulation, and quick procedure.

The evaluation of dental occlusion is crucial before indicating a cantilevered bridge. Sun et al. [13] found a 100% survival rate at 10 years for clinical situations with an open bite manifested by an overjet >0.5 mm and an overbite <1 to 1.5 mm. The cantilevered bridge has a better survival rate in cases of open bite and limited overlap.

The use of PMMA in various dental applications is mainly due to its low density, cost-effectiveness, mechanical properties, aesthetic appeal, and variety of shades. In fixed prosthodontics, PMMA is primarily used for the fabrication of temporary crowns and bridges. These materials are usually presented in powder and liquid form, either cold-cured or heat-cured. With the development of CAD/CAM techniques, pre-polymerized PMMA blocks and discs became available, allowing for polymerization away from the tooth surface and reducing the risk of residual monomers that could potentially harm oral soft tissue [9]. Studies show that CAD/CAM PMMA exhibits superior hardness and better fit compared with traditional materials. Moreover, it has improved surface properties that influence its capacity to resist bacteria and stains, making it a viable option for durable restorations.

Huettig et al. [14] showed that the longevity of CAD/CAM PMMA long-term restorations is reliable as long as the biological prognosis of the abutment teeth was acceptable. As for the longevity and stability of cantilevered bridges replacing a lateral incisor, it has been shown that the success of such restorations can be owed to the use of a single abutment tooth, a favorable occlusal context, respect of the bonding protocol, and use of etchable material.

Nonetheless, the longevity of CAD/CAM PMMA cantilevered bridges particularly is still to be confirmed by more than several case reports and clinical success stories. So far, the mechanical features of CAD/CAM PMMA found in vitro as well as the absence of residual monomer are quite promising for long-term clinical applications [15, 16].

However, CAD/CAM PMMA cantilevered bridges compared with other restorative options, such as implant-supported crowns, come with a few drawbacks. One major disadvantage is their limited durability, as wear and tear over time can lead to chipping or fracturing. Additionally, PMMA has lower strength compared with materials like ceramics and metals, making them more prone to cracking or breaking under heavy occlusal forces. Furthermore, PMMA is more prone to bacterial adhesion, staining, and color instability compared with ceramics. In addition, this type of cantilevered bridge requires healthy abutment teeth, as it relies on a single tooth for support, which means that the tooth must be healthy and strong enough to support the bridge. If the abutment tooth is compromised or too weak, the bridge may not be a suitable option [17, 18].

It is worth noting that many of these potential disadvantages can be mitigated through careful patient selection, proper maintenance, and adherence to best practices in prosthodontic treatment.

## 5. Conclusion

Cantilevered CAD/CAM PMMA bridges can be a reliable option to mid- to long-term restoration of missing upper lateral incisors. This type of restoration proved to be a quick, simple, cost-effective, and dependable solution once it is been properly indicated. However, when replacing a missing maxillary lateral incisor other factors, such as patient age, occlusion, and overall state of dentition, should influence the choice of restoration.
